# The impact of glutamate infusion on postoperative NT-proBNP in patients undergoing coronary artery bypass surgery: a randomized study

**DOI:** 10.1186/s12967-020-02351-7

**Published:** 2020-05-11

**Authors:** Huiqi Jiang, Jonas Holm, Mårten Vidlund, Farkas Vanky, Örjan Friberg, Yanqi Yang, Rolf Svedjeholm

**Affiliations:** 1 Department of Cardiothoracic Surgery, Faculty of Medicine and Health Sciences, Unit of Cardiovascular Sciences, Linköping University, Linköping, Sweden; 2 Department of Cardiothoracic Surgery, Sun Yat-Sen Memorial Hospital, Sun Yat-sen University, Guangzhou, Guangdong China; 3 Department of Cardiothoracic and Vascular Surgery, Faculty of Medicine and Health, Örebro University, Örebro, Sweden

**Keywords:** Glutamic acid, Natriuretic peptide, Heart failure, Coronary artery bypass surgery, Postoperative care

## Abstract

**Background:**

Glutamate, a key intermediate in myocardial metabolism, may enhance myocardial recovery after ischemia and possibly reduce the incidence and severity of postoperative heart failure in coronary artery bypass surgery (CABG). N-terminal pro-B-type natriuretic peptide (NT-proBNP) can be used to assess postoperative heart failure (PHF) after CABG. Our hypothesis was that glutamate enhances myocardial recovery in post-ischemic heart failure and, therefore, will be accompanied by a mitigated postoperative increase of NT-proBNP.

**Methods:**

Substudy of the GLUTAmate for Metabolic Intervention in Coronary Surgery (GLUTAMICS) trial (ClinicalTrials.gov Identifier: NCT00489827) a prospective triple-center double-blind randomized clinical trial on 399 patients undergoing CABG with or without concomitant procedure for acute coronary syndrome at three Swedish Cardiac Surgery centres (Linköping, Örebro, and Karlskrona) from May 30, 2007 to November 12, 2009. Patients were randomly assigned to intravenous infusion of 0.125 M l-glutamic acid or saline (1.65 mL/kg of body weight per hour) intraoperatively and postoperatively. Plasma NT-proBNP was measured preoperatively, the first (POD1) and third postoperative morning (POD3). A Clinical Endpoints Committee, blinded to both intervention and NT-proBNP used prespecified criteria to diagnose PHF. The primary endpoints were the absolute levels of postoperative NT-proBNP and the difference between preoperative and postoperative levels of NT-proBNP.

**Results:**

Overall no significant difference was detected in postoperative NT-proBNP levels between groups. However, in high-risk patients (upper quartile of EuroSCORE II ≥ 4.15; glutamate group n = 56; control group n = 45) glutamate was associated with significantly lower postoperative increase of NT-proBNP (POD3-Pre: 3900 [2995–6260] vs. 6745 [3455–12,687] ng•L^−1^, p = 0.012) and lower NT-proBNP POD3 (POD3: 4845 [3426–7423] vs. 8430 [5370–14,100] ng•L^−1^, p = 0.001). After adjusting for significant differences in preoperative demographics, NT-proBNP POD3 in the glutamate group was 0.62 times of that in the control group (p = 0.002). Patients in the glutamate group also had shorter ICU stay (21 [19–26] vs. 25 [22–46] h, p = 0.025) and less signs of myocardial injury (Troponin T POD3 (300 [170–500] vs. 560 [210–910] ng•L^−1^, p = 0.025).

**Conclusions:**

Post hoc analysis of postoperative NT-proBNP suggests that intravenous infusion of glutamate may prevent or mitigate myocardial dysfunction in high-risk patients undergoing CABG. Further studies are necessary to confirm these findings.

*Trial registration* Swedish Medical Products Agency 151:2003/70403 (prospectively registered with amendment about this substudy filed March 17, 2007). ClinicalTrials.gov Identifier: NCT00489827 (retrospectively registered) https://clinicaltrials.gov/ct2/show/NCT00489827?term=glutamics&draw=1&rank=1

## Background

Postoperative heart failure is the main cause for mortality after cardiac surgery [1, 2].

B-type natriuretic peptide (BNP) and N-terminal pro-B-type natriuretic peptide (NT-proBNP) are established biomarkers for diagnosis and management of heart failure in cardiology [3, 4]. Other biomarkers for heart failure such as adrenomedullin, ST2 and galectin-3 could have been considered but they were not available to us when the study was conducted. Although these biomarkers are promising NT-proBNP and BNP remain the gold standards for heart failure and they have also received more thorough evaluation in cardiac surgery. High postoperative levels of NT-proBNP and BNP are reported to be associated with need of postoperative inotropes, mechanical support and postoperative heart failure [5–10].

Glutamate plays a key role in myocardial metabolism. Although the contribution of amino acids as energy substrates is modest the qualitative role of certain amino acids is important during ischemia [11–15]. The myocardial glutamate (and aspartate) amino acid pool is critically important for anaerobic metabolism and recovery of oxidative metabolism after ischemia [12, 14, 15]. During ischemia regeneration of cytosolic NAD is essential for anaerobic glycolysis for which these amino acids play a key role through their involvement the malate-aspartate shuttle [12, 14]. Glutamate also contributes to an alternative anaerobic pathway in the mitochondria regenerating high-energy phosphates by substrate level phosphorylation in the Krebs cycle. Myocardial ischemia is associated a depletion of these amino acids and Krebs cycle intermediates [12, 15]. After ischemia glutamate contributes to replenishment of Krebs cycle intermediates lost during ischemia to enhance recovery of oxidative metabolism in postischemic hearts [12, 13, 15]. In animal models glutamate administration to ischemic or hypoxic hearts has been associated with improved maintenance of high energy phosphate levels, improved utilization of oxygen after ischemia and improved recovery of myocardial contractility [11–15]. These mechanisms seem to play an important role also in human hearts as metabolism of hearts with coronary artery disease is characterized by increased uptake of glutamate and increased release of alanine [16–18]. Early after coronary artery bypass surgery (CABG) glutamate is extracted at fraction rates only matched by oxygen suggesting increased demands [18]. Infusion of glutamate after coronary surgery increased myocardial uptake of glutamate and enhanced metabolic recovery observed as a shift from release of lactate and ammonia to their uptake. Metabolic recovery was associated hemodynamic recovery [19, 20]. In the GLUTAmate for Metabolic Intervention in Coronary Surgery GLUTAMICS-trial (ClinicalTrials.gov Identifier: NCT00489827), glutamate infusion was associated with a reduced risk of developing severe heart failure in high-risk groups [21].

Our hypothesis was that glutamate enhances myocardial recovery in post-ischemic heart failure and, therefore, will be accompanied by a mitigated postoperative increase of NT-proBNP. In this substudy of the GLUTAMICS-trial our aim was to assess the influence of intravenous glutamate infusion on postoperative NT-proBNP levels in patients undergoing CABG for acute coronary syndrome.

## Methods

### Ethics

The Regional Ethical Review Board in Linköping, Sweden (original protocol no M76-05; addendum 26-07) approved this substudy of the GLUTAMICS trial. The study was conducted according to the Helsinki Declaration of Human Rights and all patients were enrolled in the study after written informed consent.

### Patients

**Fig. 1:**
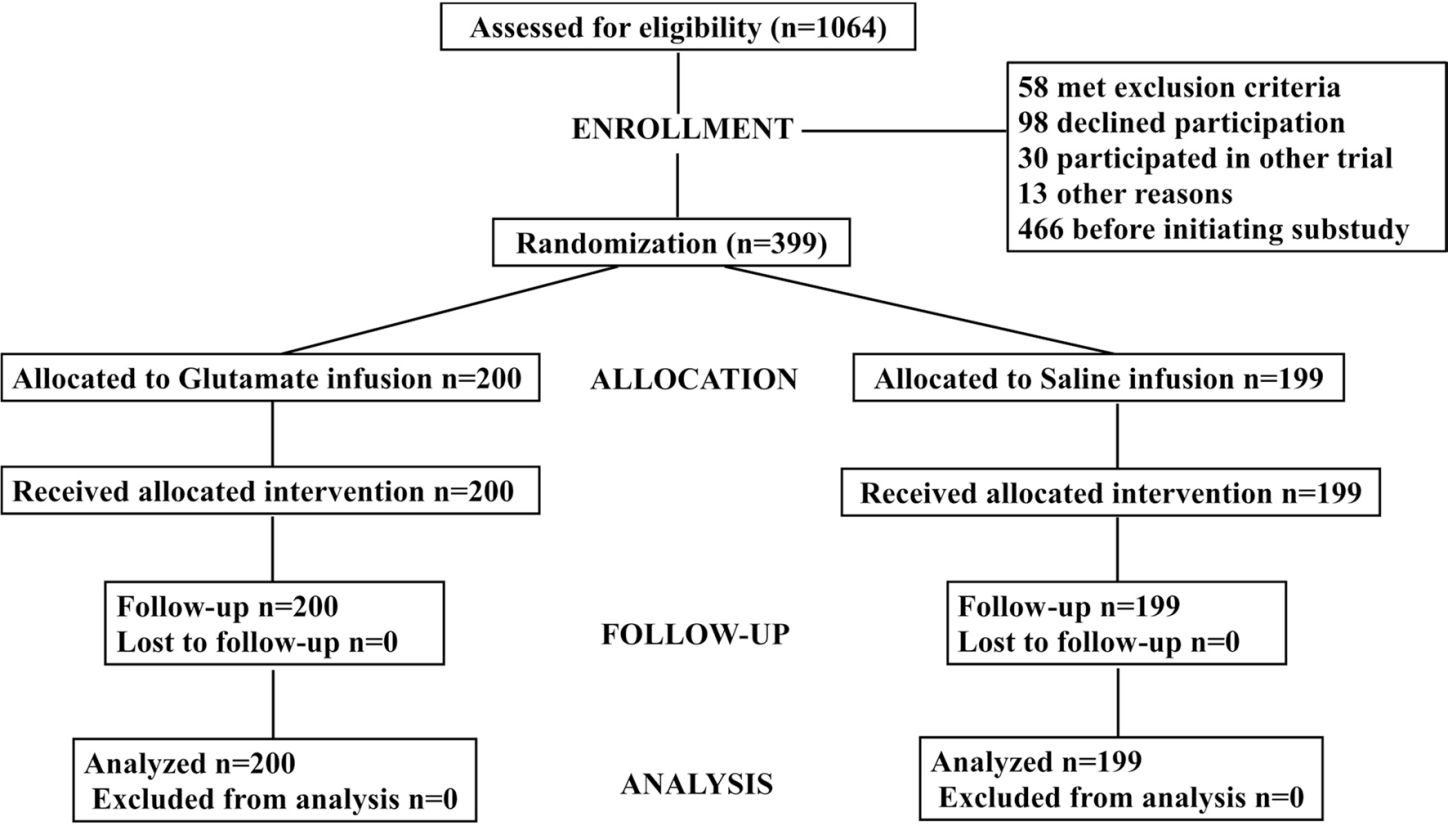
Flow chart of the patients

This substudy was initiated after amendments to the Swedish Medical Product Agency and the Regional Ethical Review Board when NT-proBNP became available to the investigators of the GLUTAMICS. The study population consisted of 399 consecutive patients from the GLUTAMICS trial with acute coronary syndrome undergoing urgent CABG with or without concomitant valve procedure at three Swedish Cardiac Surgery centres (Linköping, Örebro, and Karlskrona) from May 30, 2007 to November 12, 2009 [21]. Originally powered for 2214 patients with regard to the intervention the trial was terminated per protocol after interim analysis because of prespecified stopping criteria [21]. A total of 1064 patients with acute coronary syndrome, eligible for CABG intervention, were assessed and 861 patients were included in the original study [21]. A flow chart of the patients in the present study is given in Fig. 1. Sample size was thus limited by the volume of patients with NT-proBNP measurements in the GLUTAMICS-trial. Details of number of patients with NT-proBNP samples at different time points are given in the results section.

### Study design

This is a prespecified substudy of an investigator-initiated, prospective, double-blind randomized clinical trial, the GLUTAMICS-trial to assess the influence of intravenous glutamate infusion on postoperative NT-proBNP levels in patients undergoing CABG for acute coronary syndrome [21]. The patients were randomly allocated to blinded intravenous infusion of 0.125 M l-glutamic acid or saline at a rate of 1.65 mL/kg of body weight per hour beginning at the induction of anesthesia. The infusion was temporarily stopped during cardioplegic arrest and resumed after declamping the aorta for an additional 2 h, after which the infusion rate was halved and an additional 50 mL infused. The maximum volume infused to any patient did not exceed 500 mL of study solution. The concentration of the solution was determined by the solubility of l-glutamic acid and the dosage of glutamate was based on data showing that increase of arterial whole blood levels by two- to threefold was sufficient to saturate the myocardial capacity to extract glutamate from the circulation early after CABG [22]. For further details regarding the GLUTAMICS-trial and the glutamate solution see Additional file 1 or the original publication [21].

Post-hoc analysis of high-risk patients belonging to the upper quartile according to EuroSCORE II (≥ 4.15) and patients treated with inotropes was also performed.

Venous blood samples for NT-proBNP were drawn at three time points: before induction of anesthesia, the first and third morning after surgery. NT-proBNP in plasma was measured with electro-chemoiluminescence immunoassay on a Roche Elecsys 2010 automated platform (Roche Diagnostics, Basel, Switzerland). The assay had an effective measuring range of 5–35,000 ng/L. The inter-assay coefficient of variation was at 175 ng/L CV = 2.7%, 355 ng/L CV = 2.4% and 1068 ng/L CV = 1.9%. The results of the assays were released from the laboratory when the trial was terminated. An independent professional monitoring team (Clinical Research Support Örebro, Sweden) performed external monitoring of all key study data (correct inclusion, signed informed consent, serious adverse events, suspected unexpected serious adverse reactions, stroke, mortality, troponin-T, CK-MB, electrocardiogram, and the criteria used for PHF and severe PHF).

### Study endpoints

The primary endpoints were the absolute levels of postoperative NT-proBNP and the difference between preoperative and postoperative levels of NT-proBNP.

### Clinical management

Clinical management was standardized and similar at the three participating centres with minor differences concerning choice of anesthetic drugs. Standard surgical techniques were used. In 387 patients standard use of cardiopulmonary bypass (CPB) and aortic cross-clamping was employed. Cold blood cardioplegia was used for myocardial protection in 81% of the patients whereas crystalloid cardioplegia was used in the remaining patients operated on pump. Twelve patients were operated off pump.

A surgical pulmonary artery catheter was introduced intraoperatively in all patients for measurement of mixed venous oxygen saturation (SvO_2_) and pulmonary artery pressures [23]. Transesophageal echocardiography was routinely employed.

After discharge from the ICU, patients were transferred to a step-down semi-intensive care unit for at least 24 h before going to the general ward. Further details on clinical management can be found in Additional file 1.

### Definitions

#### Postoperative heart failure

Patients were considered to have PHF if criteria a + b were fulfilled.

- Decision reached by the Endpoints committee that heart failure was evident at weaning from cardiopulmonary bypass or during the early hours after surgery based on criteria below and supported by available clinical records, echocardiography and hemodynamic data.
- SvO_2_ criteria in relation to systolic arterial pressure (SAP) that could not be explained by shivering, anemia or hypovolemia. The criteria were based on extensive studies on SvO_2_ with regard to outcome and clinical experience regarding the relationship between SvO_2_ and SAP while using fast acting vasodilators [23–26].

- SvO_2_ < 50% and SAP < 130 mmHg
- SvO_2_ < 55% and SAP < 110 mmHg
- SvO_2_ < 60% and SAP < 90 mmHg
- SvO_2_ < 65% and SAP < 70 mmHg

#### Severe postoperative heart failure

Severe postoperative heart failure was defined as PHF associated with death or requiring treatment with intra-aortic balloon pump or need for at least one inotropic agent in dosages listed in the Additional file 1 ≥ 24 h admission to ICU in patients requiring extended ICU stay (≥ 48 h). Further details are given in Additional file 1.

### Hospital mortality

Hospital mortality was defined as mortality during the first hospitalisation period including stay at the referral hospital after discharge from the cardiac surgical unit. Cardiac cause of death was defined as death caused by or initiated by a cardiac event such as heart failure or myocardial infarction.

### Myocardial injury

Myocardial injury was measured with Creatine Kinase-MB isoenzyme (CK-MB) on the first postoperative morning and with Troponin T on the third postoperative day. The different time points for sampling were chosen with regard to the different release kinetics of these biomarkers in permanent myocardial injury [27].

### Acute kidney injury

Acute kidney injury (AKI) was defined as a postoperative increase of plasma-Creatinine by 50% or more compared with preoperative level in accordance with RIFLE-criteria.

### Postoperative stroke

Postoperative stroke was defined as neurological or cognitive deficit with a cerebral injury verified on (Computed Tomography) CT-scan. All suspected cases of stroke underwent CT-scan.

### Left ventricular dysfunction

Moderate left ventricular dysfunction corresponds to an ejection fraction of 0.45–0.31 according to echocardiography. Severe left ventricular dysfunction corresponds to an ejection fraction of 0.30 or less.

### Statistical analysis

Categorical variables are presented as percentages and continuous variables that were not normally distributed are expressed as medians with interquartile range. To minimize data loss, missing data were managed with pairwise deletion when possible. Fisher’s exact test was used to compare categorical data. Mann–Whitney U test was used to compare continuous variables not following a normal distribution.

NT-proBNP was log_10_ transformed before linear regression analysis because of its skewed distribution. A multivariable linear regression model with regard to log10 postoperative NT-proBNP was used to assess the role of glutamate on postoperative NT-proBNP level, adjusting for preoperative and intraoperative variables that differed significantly between glutamate group and control group. A p-value < 0.05 was considered to be statistically significant; all p-values were two-sided.

Statistical analyses were performed with SPSS statistics version 23 (IBM) for windows.

## Results

A total of 399 patients (glutamate group n = 200, control group n = 199) patients undergoing CABG for acute coronary syndrome had at least one available NT-proBNP measurement as follows: preoperative (n = 383), postoperative day 1 (n = 334) and postoperative day 3 (n = 339). A complete set of NT-proBNP data was available at all time points in 280 patients; Preop and POD1 in 320 patients; Preop and POD3 in 325 patients. The median age was 69 [63–75] years and 19% were female. In 17 patients CABG with a concomitant procedure was done (mitral valve surgery n = 8, aortic valve surgery n = 7, and ablation for atrial fibrillation n = 2). The median EuroSCORE II was 2.44 [1.65–4.15]. Postoperative heart failure developed in 40 patients and 10 of these developed severe circulatory failure. Preoperative, intraoperative and postoperative characteristics of the 399 patients are presented in Table 1.

**Table 1:** Preoperative characteristics of all patients, the glutamate group and the control group (saline)

Variables	All patients (n = 399)	Glutamate (n = 200)	Control (n = 199)	p-value
Age (years)	69 [63–75]	69 [63–76]	68 [62–75]	0.45
Female gender	19% (77)	17% (34)	22% (43)	0.26
BMI (kg•m^2^)	27 [24–30]	26 [24–76]	27 [25–30]	0.18
EuroSCORE II	2.44 [1.66–4.15]	2.52 [1.72–4.41]	2.35 [1.61–3.89]	0.42
Diabetes	23% (93)	21% (41)	26% (52)	0.19
Hypertension	60% (240)	62% (123)	59% (117)	0.54
COPD	7% (27)	9% (17)	5% (10)	0.23
NT-proBNP (ng•L^−1^)	440 [150–1017]	470 [185–1070]	420 [140–999]	0.31
Hemoglobin (g•L^−1^)	139 [129–147]	138 [128–146]	139 [130–149]	0.58
Troponin T (ng•L^−1^)	0 [0–60]	0 [0–50]	10[0–70]	0.46
p-Creatinine (μmol•L^−1^)	91 [79–104]	92 [81–106]	90 [78–104]	0.24
eGFR (mL•min^−1^•1.73 m^−2^)	76 [58–97]	73 [57–95]	77 [59–100]	0.23
Cerebrovascular disease	9% (34)	8% (16)	9% (18)	0.72
Three-vessel disease	77% (306)	76% (152)	77% (154)	0.81
Left main stenosis	36% (145)	39% (78)	34% (67)	0.3
AMI < 3 weeks	65% (260)	67% (134)	63% (126)	0.46
History of AMI	73% (291)	75% (150)	71% (141)	0.37
CCS IV	60% (239)	59% (119)	61% (122)	0.61
Angina at rest < 48 h preoperatively	16% (62)	14% (27)	18% (35)	0.27
Moderate LV dysfunction	13% (52)	14% (28)	12% (24)	0.66
Severe LV dysfunction	4% (16)	3% (5)	6% (11)	0.14

Data given as medians [interquartile range] or percentages (number)

AMI < 3 weeks: acute myocardial infarction within 3 weeks of surgery; Angina at rest < 48 h preoperatively: angina at rest within the last 48 h before surgery

*BMI* body mass index, *CCS* Canadian Cardiovascular Society, *COPD* chronic obstructive pulmonary disease, *EuroSCORE II* European system for cardiac operative risk evaluation II, *LV* left ventricular, *eGFR* estimated glomerular filtration rate according to MDRD formula

Preoperative, intraoperative and postoperative data did not differ significantly between the glutamate group and the control group (Tables 1 and 2).

**Table 2:** Intraoperative and postoperative characteristics of all patients, the glutamate group and the control group (saline)

Variables	All patients (n = 399)	Glutamate (n = 200)	Control (n = 199)	p-value
Aortic crossclamp time (min)	50 [40–64]	53 [39–63]	48 [40–65]	0.95
CPB time (min)	76 [63–97]	77 [62–97]	76 [64–97]	0.94
Reperfusion time (min)	21 [15–29]	20 [16–30]	21 [15–28]	0.49
NT-proBNP POD1 (ng•L^−1^)	2175 [1340–3770]	2220 [1484–4040]	2041 [1236–3429]	0.18
NT-proBNP POD3 (ng•L^−1^)	3690 [2239–6065]	3640 [2335–6155]	3781 [2081–6020]	0.95
Delta NT-proBNP POD1-Pre (ng•L^−1^)	1590 [1029–2725]	1640 [1069–2801]	1522 [910–2640]	0.49
Delta NT-proBNP POD3-Pre (ng•L^−1^)	3067 [1617–5038]	2995 [1671–5009]	3110 [1580–5297]	0.94
CK-MB POD1 (µg•L^−1^)	15 [10–23]	14 [10–23]	15 [11–23]	0.43
TroponinT POD3 (ng•L^−1^)	250 [140–510]	260 [150–490]	250 [140–550]	0.87
Delta troponin T POD3-Pre (ng•L^−1^)	190 [95–385]	195 [90–390]	180 [100–370]	0.84
Inotropic support	24% (95)	28% (56)	20% (39)	0.06
ICU stay (h)	21 [17–23]	20 [17–23]	21 [17–24]	0.21
ICU stay > 72 h	6% (23)	6% (12)	6% (11)	1
Ventilation time (h)	4 [3–6]	4 [3–6]	4 [3–6]	0.77
Ventilation time > 48 h	3% (11)	3% (6)	3% (5)	1
PHF	10% (40)	10% (19)	11% (21)	0.74
Severe PHF	3% (10)	2% (3)	4% (7)	0.22
Postoperative stroke	2% (7)	2% (4)	2% (3)	1
AKI	15% (61)	17% (33)	14% (28)	0.58
Hospital mortality	1% (5)	1% (2)	2% (3)	0.69

Data given as medians [interquartile range] or percentages (number)

*AKI* acute kidney injury, *CK-MB* creatine kinase-MB isoenzyme, *CPB* cardiopulmonary bypass, *ICU* intensive care unit, *POD* postoperative day

### Influence of glutamate on postoperative NT-proBNP

In the whole cohort, postoperative NT-proBNP levels did not differ significantly between the glutamate group and the control group (POD1: 2220 [1484–4040] vs. 2041 [1236–3429] ng•L^−1^, p = 0.18; POD3: 3640 [2335–6155] vs. 3781 [2081–6020] ng•L^−1^, p = 0.95).

### Influence of glutamate on postoperative NT-proBNP in patients with increased preoperative risk (EuroSCORE II ≥ 4.15)

Post hoc analysis was done on 101 patients in the upper quartile of risk according to preoperative EuroSCORE II ≥ 4.15 (glutamate group n = 56; control group n = 45). The perioperative characteristics for patients with EuroSCORE II ≥ 4.15 are given in Tables 3 and 4. The groups were evenly distributed, with the exception of higher preoperative Troponin T (40 [0–390] vs. 0 [0–30] ng•L^−1^, p < 0.0001) and more patients with angina at rest less than 48 h before surgery (36% vs. 13%, p = 0.007) in the control group.

**Table 3:** Preoperative characteristics in patients with EuroSCORE II ≥ 4.15

Variables	EuroSCORE II≥ 4.15 (n = 101)	Glutamate (n = 56)	Control (n = 45)	p-value
Age (years)	76 [71–79]	76 [70–79]	76 [72–79]	0.85
Female gender	38% (38)	32% (18)	44% (20)	0.22
BMI (kg•m^2^)	25 [22–28]	25 [22–28]	26 [23–28]	0.64
EuroSCORE II	5.83 [4.89–7.85]	5.86 [5.00–7.54]	5.83 [4.54–8.97]	0.82
Diabetes	29% (29)	27% (14)	33% (15)	0.38
Hypertension	72% (72)	68% (38)	76% (34)	0.50
COPD	18% (18)	21% (12)	13% (6)	0.31
NT-proBNP (ng•L^−1^)	1010 [450–2345]	790 [425–1895]	1265 [465–2915]	0.15
Hemoglobin (g•L^−1^)	132 [121–142]	133 [125–143]	130 [115–139]	0.08
Troponin T (ng•L^−1^)	10 [0–90]	0 [0–30]	40 [0–390]	< 0.0001
p-Creatinine (μmol•L^−1^)	97 [88–122]	97 [87–115]	97 [90–123]	0.51
eGFR (mL•min^−1^•1.73 m^−2^)	50 [41––68]	50 [44–72]	51 [38–66]	0.38
Cerebrovascular disease	21% (21)	20% (11)	23% (10)	0.81
Three-vessel disease	83% (83)	82% (46)	82% (37)	1
Left main stenosis	48% (48)	45% (25)	51% (23)	0.55
AMI < 3 weeks	73% (73)	73% (41)	71% (32)	0.83
History of AMI	82% (82)	82% (46)	80% (36)	0.80
CCS IV	80% (80)	82% (46)	76% (34)	0.47
Angina at rest < 48 h preoperatively	23% (23)	13% (7)	36% (16)	0.007
Moderate LV dysfunction	21% (21)	23% (13)	18% (8)	0.62
Severe LV dysfunction	14% (14)	9% (5)	20% (9)	0.15

Data given as medians [interquartile range] or percentages (number)

AMI < 3 weeks: acute myocardial infarction within 3 weeks of surgery; Angina at rest < 48 h preoperatively: angina at rest within 48 h before surgery

*BMI* body mass index, *CCS*: Canadian Cardiovascular Society, *COPD* chronic obstructive pulmonary disease, *EuroSCORE II* European system for cardiac operative risk evaluation II, *LV* left ventricular eGFR: estimated glomerular filtration rate according to MDRD formula

**Table 4:** Intraoperative and postoperative characteristics in patients with EuroSCORE II ≥ 4.15

Variables	EuroSCOREII ≥ 4.15 (n = 101)	Glutamate (n = 56)	Control (n = 45)	p-value
Aortic crossclamp time (min)	53 [42–67]	54 [41–65]	52 [42–67]	0.91
CPB time (min)	80 [65–106]	81 [64–104]	79 [67–107]	0.74
Reperfusion time (min)	23 [16–30]	24 [17–30]	21 [16–29]	0.53
NT-proBNP POD1 (ng•L^−1^)	4170 [2850–6185]	3786 [2630–5380]	4675 [3010–6650]	0.21
NT-proBNP POD3 (ng•L^−1^)	6650 [3800–9970]	4845 [3426–7423]	8430 [5370–14,100]	0.001
Delta NT-proBNP POD1-Pre (ng•L^−1^)	2800 [1650–4760]	2653 [1540–3675]	3050 [2030–5560]	0.13
Delta NT-proBNP POD3-Pre (ng•L^−1^)	5210 [3115–8595]	3900 [2995–6260]	6745 [3455–12,687]	0.012
CK-MB POD1 (µg•L^−1^)	18 [11–25]	14 [9–23]	19 [12–26]	0.12
TroponinT POD3 (ng•L^−1^)	390 [190–730]	290 [160–480]	550 [210–860]	0.015
Delta Troponin T POD3-Pre (ng•L^−1^)	270 [135–600]	270 [130–450]	350 [150–680]	0.44
Inotropic support	43% (43)	43% (24)	42% (19)	1.00
ICU stay (h)	22 [19–42]	21 [19–26]	25 [22–45]	0.022
ICU stay > 72 h	18% (18)	16% (9)	20% (9)	0.61
Ventilation time (h)	5 [3–12]	4 [3–9]	5 [3–20]	0.3
Ventilation time > 48 h	11% (11)	11% (6)	11% (5)	1
PHF	22% (22)	15% (8)	31% (14)	0.053
Severe PHF	9% (9)	4% (2)	16% (7)	0.074
Postoperative stroke	4% (3)	4% (2)	4% (2)	1
AKI	29% (29)	25% (14)	33% (15)	0.51
Hospital Mortality	5% (5)	4% (2)	7% (3)	0.65

Data given as medians [interquartile range] or percentages (number)

*AKI* acute kidney injury, *CK-MB* creatine kinase-MB isoenzyme, *CPB* cardiopulmonary bypass, *ICU* intensive care unit, *PHF* postoperative heart failure, *POD* postoperative day

**Fig. 2:**
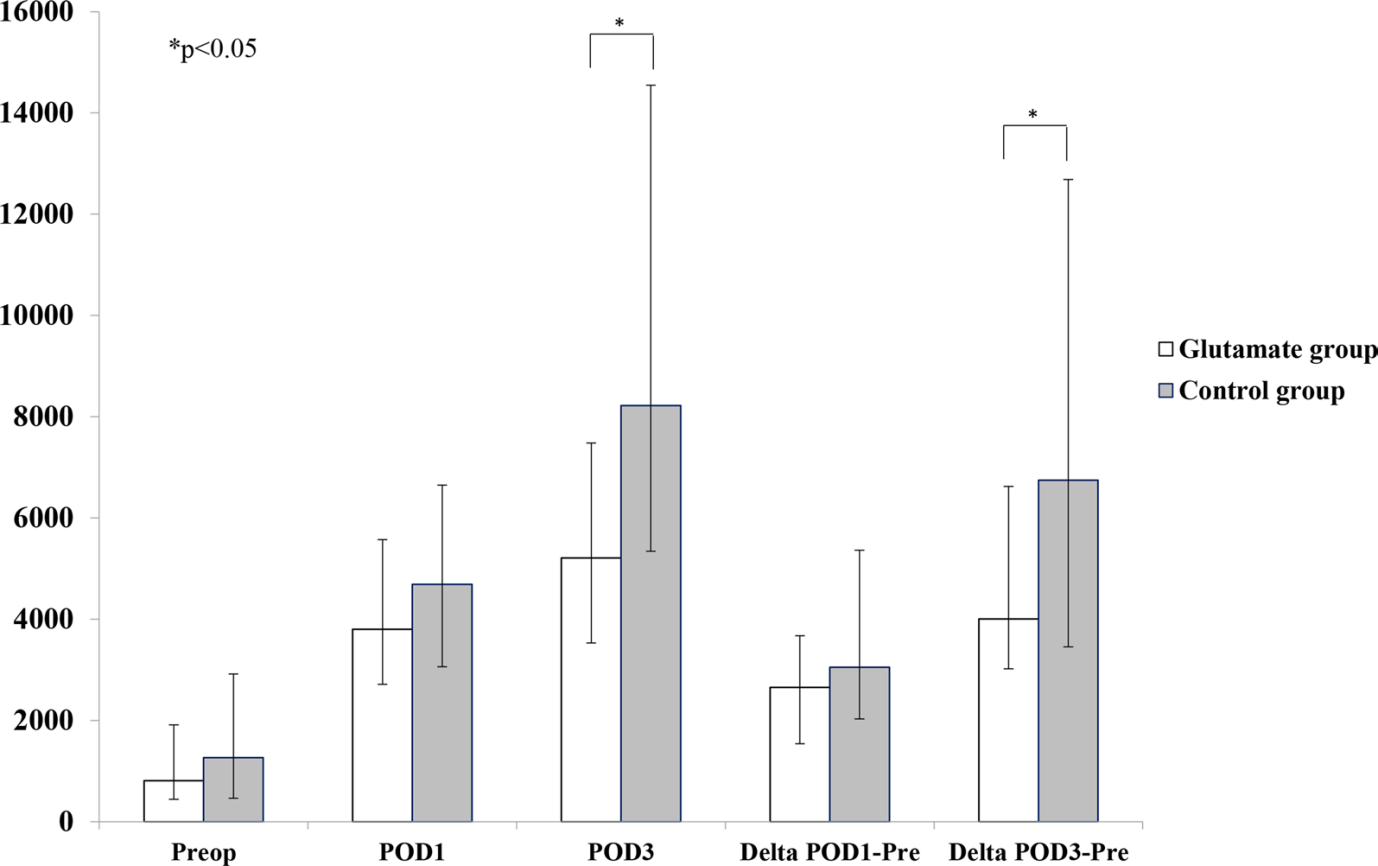
Perioperative NT-proBNP levels in patients with EuroSCORE II ≥ 4.15 (upper quartile). Data expressed as medians with interquartile range. *p < 0.05. *POD* postoperative day, *Preop* preoperative

In the glutamate group patients had significantly lower postoperative increase of NT-proBNP (POD3-Pre: 3900 [2995–6260] vs. 6745 [3455–12,687] ng•L^−1^, p = 0.012,) and lower absolute levels of NT-proBNP POD3 compared to the control group (POD3: 4845 [3426–7423] vs. 8430 [5370–14,100] ng•L^−1^, p = 0.001) (Table 4, Fig. 2). Similar results were also found if only patients with complete data sets of NT-proBNP were analyzed (Table 5).

**Table 5:** Perioperative NT-proBNP in patients with complete data sets of NT-proBNP Preop and POD1 or NT-proBNP Preop and POD3 and EuroSCORE II ≥ 4.15

Variables	EuroSCORE II ≥ 4.15 (n = 81^a^/83^b^)	Glutamate (n = 41^a^/47^b^)	Control (n = 37^a^/36^b^)	p-value
NT-proBNP Pre^a^ (ng•L^−1^)	1090 [460–2430]	980 [435–2057]	1340 [460–2910]	0.38
NT-proBNP POD1^a^ (ng•L^−1^)	4390 [2950–6300]	3802 [2715–5570]	4690 [3060–6650]	0.19
Delta NT-proBNP POD1-Pre^a^ (ng•L^−1^)	2800 [1650–4760]	2653 [1540–3675]	3050 [2030–5560]	0.13
NT-proBNP Pre^b^ (ng•L^−1^)	970 [425–2345]	740 [395–1810]	1265 [465–2985]	0.10
NT-proBNP POD3^b^ (ng•L^−1^)	6650 [3840–10,085]	5140 [3490–7423]	8220 [5339–14,550]	0.002
Delta NT-proBNP POD3-Pre^b^ (ng•L^−1^)	5210 [3115–8595]	3900 [2995–6260]	6745 [3455–12,686]	0.012

Data given as medians [interquartile range] or percentages (number)

*Preop* preoperative, *POD* postoperative day

^a^Patients with complete data sets of NT-proBNP Preop and POD1

^b^Patients with complete data sets of NT-proBNP Preop and POD3

After adjusting for preoperative Troponin T and incidence of angina at rest less than 48 h before surgery, only glutamate remained in the final multivariable linear regression model with regard to log_10_NT-proBNP POD3. NT-proBNP POD3 in the glutamate group was 0.62 times of that in the control group (adjusted coefficient − 0.208, 95%CI − 0.336 to − 0.080; p = 0.002). Type of myocardial protection did not influence the results.

Postoperatively patients in the glutamate group had less signs of myocardial injury (Troponin T POD3: 290 [160–480] vs. 550 [210–860] ng•L^−1^, p = 0.015), shorter ICU stay (21 [19–26] vs. 25 [22–45] h, p = 0.022) and a trend towards lower incidence of PHF (14%, n = 8 vs. 31%, n = 14, p = 0.053) and severe PHF (4%, n = 2 vs. 16%, n = 7, p = 0.074) (Table 4).

### NT-proBNP in patients treated with inotropes

Patients treated with inotropes had substantially higher NT-proBNP levels both preoperatively and postoperatively at all time points (Additional file 1: Table S1). However, patients in the glutamate group had significantly lower increases of NT-proBNP postoperatively and significantly lower absolute levels of NT-proBNP on POD3 (Additional file 1: Tables S2, S3).

## Discussion

In this substudy of the GLUTAMICS trial we were unable to detect an effect of glutamate on postoperative NT-proBNP levels in the whole cohort. However, in a post hoc analysis of patients belonging to the upper quartile of operative risk glutamate was associated with less increase of NT-proBNP from preoperative level to POD3 and significantly lower absolute levels on POD3. In patients treated with inotropes glutamate was associated with less increase of NT-proBNP and significantly lower levels on POD3. To our knowledge, this is the first study to evaluate the impact of glutamate on NT-proBNP in patients undergoing CABG.

Glutamate plays a key role in myocardial metabolism in particular during myocardial ischemia [11, 12, 14, 15]. The contractile function of the heart is immediately and inextricably linked to substrate metabolism [28]. As glutamate has no intrinsic functional effect on the myocardium and we have been unable to detect a significant vasodilator effect with the used solution and infusion rate its effect is dependent on whether there is a metabolic derangement which glutamate can facilitate the recovery of [19, 20, 29, 30]. As described in detail in the introduction glutamate could act in two different ways. Firstly, glutamate might improve myocardial tolerance to ischemia during ongoing ischemia by facilitating anaerobic metabolism in the cytosol through its role in the malate-aspartate shuttle and by substrate level phosphorylation in the mitochondria [11, 12, 14, 15]. Secondly, glutamate could enhance recovery of myocardial oxidative metabolism after ischemia by replenishment of Krebs cycle intermediates [12–15]. The first mechanism has been demonstrated in animal experiments and in patients with angina but has been difficult to show in the setting of cardiac surgery [11, 12, 14, 15, 31]. A possible reason might be that myocardial infarcts in association with CABG often are caused by ischemia preoperatively just before surgery or during the early stages of surgery [32]. The second mechanism, enhancement of post-ischemic recovery of myocardial metabolism has been shown in patients with proven metabolic disturbances [19, 20]. As might be expected such an effect could not be detected in low risk patients with no or minimal metabolic derangement [33].

The impact of glutamate infusion on functional recovery of the heart thus seems to be related to the magnitude of metabolic derangement before treatment [30]. In the GLUTAMICS-trial, a high proportion of low risk patients were included [21]. Glutamate administration contributes little to these patients as the high myocardial extraction rate of glutamate from blood seen normally early after CABG is sufficient for recovery of myocardial metabolism in most of these patients [18]. This might explain why there was no evident the impact of glutamate on postoperative NT-proBNP in the whole cohort.

High plasma concentrations of BNP and NT-proBNP postoperatively are associated with increased use of inotropes and or intra-aortic balloon pump in cardiac surgery [5–9, 34, 35]. Adjusting for significant differences in preoperative demographics NT-proBNP on POD3 was substantially lower in the glutamate group compared to controls. Glutamate may thus have influenced postoperative NT-proBNP levels by facilitating post-ischemic recovery of myocardial oxidative metabolism in line with our hypothesis. Undeniably though post hoc analyses preclude any firm conclusions.

Not unexpectedly, the results are in accordance with the main results of the GLUTAMICS-trial, which failed to prove an effect in the whole cohort on the composite primary endpoint, which were postoperative mortality, postoperative myocardial infarct and left ventricular heart failure on weaning from CPB. Originally the GLUTAMICS trial was planned for patients requiring urgent surgery because of CCS class IV angina but, since only three Swedish centers participated, the inclusion criteria were widened to include all patients with acute coronary syndrome. This implied that a large proportion of the included patients were low risk patients scheduled for urgent surgery because of non-ST-segment elevation myocardial infarction (NSTEMI) [21]. However, secondary endpoints and post hoc analyses found a relative risk reduction exceeding 50% for developing severe postoperative heart failure in most high-risk groups undergoing isolated CABG with the exception diabetics [21]. A subsequent post hoc analysis demonstrated that the number of inotropes needed and the duration of treatment was shorter in glutamate treated patients with heart failure at weaning from CPB [29]. The current analysis supports those findings by demonstrating that glutamate was associated with a mitigated increase and lower postoperative NT-proBNP levels on POD3 in patients treated with inotropes (Additional file 1: Tables S2, S3).

Certain study limitations deserve comment. The major limitation is that the significant results of this study were evident only in our post hoc analyses of risk patients. Furthermore, the impact of glutamate on NT-proBNP POD3 among the patients with EuroSCORE II ≥ 4.15 should be interpreted cautiously due to relatively small sample size. The study did not include specific assessment of myocardial contractility or metabolism. On the other hand, the results fit available knowledge about glutamate in myocardial metabolism and potential mechanisms of action. Further assessment of glutamate is warranted, particularly given the risks associated with inotropic treatment of the ischemic heart and the role of postoperative heart failure or low cardiac output syndrome as the major cause for morbidity and mortality in cardiac surgery [1, 2, 36–38].

## Conclusions

Our study on perioperative NT-proBNP changes confirms previous findings that patient selection is a factor that could influence the impact of metabolic treatment. Assessment of postoperative NT-proBNP suggests that intravenous infusion of glutamate may prevent or mitigate myocardial dysfunction in high-risk patients undergoing CABG. Further studies are necessary to confirm these findings.

## Supplementary information

**Additional file 1.** List additional methods and results on NT-proBNP in patients treated with inotropes related to glutamate (Table S1–S3).

## Data Availability

The datasets analysed during the current study are available from the corresponding author on reasonable request provided that professional secrecy applies.

## Abbreviations

AKI: Acute kidney injury
AMI: Acute myocardial infarction
AUC: Area under the curve
BMI: Body mass index
BNP: B-type natriuretic peptide
CABG: Coronary artery bypass surgery
CCS: Canadian Cardiovascular Society
CI: Confidence interval
CK-MB: Creatine kinase-MB isoenzyme
COPD: Chronic obstructive pulmonary disease
CPB: Cardiopulmonary bypass
eGFR: Estimated glomerular filtration rate according to MDRD formula
EuroSCORE II: European system for cardiac operative risk evaluation II
GLUTAMICS: GLUTAmate for Metabolic Intervention in Coronary Surgery trial
ICU: Intensive care unit
LV: LEFT ventricular
NT-proBNP: N-terminal pro-B-type natriuretic peptide
NSTEMI: Non-ST-segment elevation myocardial infarction
PHF: Postoperative heart failure
POD: Postoperative day
ROC: Receiver operating characteristic analysis
SAP: Systolic arterial blood pressure
SvO_2_: Mixed venous oxygen saturation
URL: Upper reference limit

## References

[1] O’Connor GT, Birkmeyer JD, Dacey LJ, Quinton HB, Marrin CA, Birkmeyer NJ, Morton JR, Leavitt BJ, Maloney CT, Hernandez F, Clough RA, Nugent WC, Olmstead EM, Charlesworth DC, Plume SK (1998). Results of a regional study of modes of death associated with coronary artery bypass grafting. Northern New England Cardiovascular Disease Study Group. Ann Thorac Surg..

[2] Surgenor SD, O’Connor GT, Lahey SJ, Quinn R, Charlesworth DC, Dacey LJ, Clough RA, Leavitt BJ, Defoe GR, Fillinger M, Nugent WC, Northern New England Cardiovascular Disease Study G (2001). Predicting the risk of death from heart failure after coronary artery bypass graft surgery. Anesth Analg..

[3] Ponikowski P, Voors AA, Anker SD, Bueno H, Cleland JG, Coats AJ, Falk V, Gonzalez-Juanatey JR, Harjola VP, Jankowska EA, Jessup M, Linde C, Nihoyannopoulos P, Parissis JT, Pieske B, Riley JP, Rosano GM, Ruilope LM, Ruschitzka F, Rutten FH, van der Meer P, Authors/Task Force M, Document R (2016). 2016 ESC guidelines for the diagnosis and treatment of acute and chronic heart failure: the task force for the diagnosis and treatment of acute and chronic heart failure of the European Society of Cardiology (ESC). Developed with the special contribution of the Heart Failure Association (HFA) of the ESC. Eur J Heart Fail..

[4] Yancy CW, Jessup M, Bozkurt B, Butler J, Casey DE, Colvin MM, Drazner MH, Filippatos GS, Fonarow GC, Givertz MM, Hollenberg SM, Lindenfeld J, Masoudi FA, McBride PE, Peterson PN, Stevenson LW, Westlake C (2017). 2017 ACC/AHA/HFSA focused update of the 2013 ACCF/AHA guideline for the management of heart failure: a report of the American College of Cardiology/American Heart Association task force on clinical practice guidelines and the Heart Failure Society of America. J Card Fail..

[5] Reyes G, Fores G, Rodriguez-Abella RH, Cuerpo G, Vallejo JL, Romero C, Pinto A (2005). NT-proBNP in cardiac surgery: a new tool for the management of our patients?. Interact Cardiovasc Thorac Surg..

[6] Fox AA, Shernan SK, Collard CD, Liu KY, Aranki SF, DeSantis SM, Jarolim P, Body SC (2008). Preoperative B-type natriuretic peptide is as independent predictor of ventricular dysfunction and mortality after primary coronary artery bypass grafting. J Thorac Cardiovasc Surg.

[7] Nozohoor S, Nilsson J, Luhrs C, Roijer A, Algotsson L, Sjogren J (2009). B-type natriuretic peptide as a predictor of postoperative heart failure after aortic valve replacement. J Cardiothorac Vasc Anesth.

[8] Suttner S, Boldt J, Lang K, Rohm KD, Piper SN, Mayer J (2008). Association of N-terminal pro-brain natriuretic peptide and cardiac troponin T with in-hospital cardiac events in elderly patients undergoing coronary artery surgery. Eur J Anaesthesiol.

[9] Provenchere S, Berroeta C, Reynaud C, Baron G, Poirier I, Desmonts JM, Iung B, Dehoux M, Philip I, Benessiano J (2006). Plasma brain natriuretic peptide and cardiac troponin I concentrations after adult cardiac surgery: association with postoperative cardiac dysfunction and 1-year mortality. Crit Care Med.

[10] Kerbaul F, Collart F, Giorgi R, Oddoze C, Lejeune PJ, Guidon C, Caus T, Bellezza M, Gouin F (2004). Increased plasma levels of pro-brain natriuretic peptide in patients with cardiovascular complications following off-pump coronary artery surgery. Intensive Care Med.

[11] Rau EE, Shine KI, Gervais A, Douglas AM, Amos EC (1979). Enhanced mechanical recovery of anoxic and ischemic myocardium by amino acid perfusion. Am J Physiol.

[12] Pisarenko OI (1996). Mechanisms of myocardial protection by amino acids: facts and hypotheses. Clin Exp Pharmacol Physiol.

[13] Lazar HL, Buckberg GD, Manganaro AJ, Becker H, Maloney JV (1980). Reversal of ischemic damage with amino acid substrate enhancement during reperfusion. Surgery..

[14] Burns AH, Reddy WJ (1978). Amino acid stimulation of oxygen and substrate utilization by cardiac myocytes. Am J Physiol.

[15] Pisarenko OI, Oleynikov OD, Shulzhenko VS, Studneva IM, Ryff IM, Kapelko VI (1989). Association of myocardial glutamate and aspartate pool and functional recovery of postischemic heart. Biochem Med Metab Biol.

[16] Mudge GH, Mills RM, Taegtmeyer H, Gorlin R, Lesch M (1976). Alterations of myocardial amino acid metabolism in chronic ischemic heart disease. J Clin Investig.

[17] Thomassen AR, Nielsen TT, Bagger JP, Henningsen P (1983). Myocardial exchanges of glutamate, alanine and citrate in controls and patients with coronary artery disease. Clin Sci.

[18] Svedjeholm R, Ekroth R, Joachimsson PO, Ronquist G, Svensson S, Tyden H (1991). Myocardial uptake of amino acids and other substrates in relation to myocardial oxygen consumption four hours after cardiac operations. J Thorac Cardiovasc Surg.

[19] Svedjeholm R, Vanhanen I, Hakanson E, Joachimsson PO, Jorfeldt L, Nilsson L (1996). Metabolic and hemodynamic effects of intravenous glutamate infusion early after coronary operations. J Thorac Cardiovasc Surg.

[20] Pisarenko OI, Lepilin MG, Ivanov VE (1986). Cardiac metabolism and performance during l-glutamic acid infusion in postoperative cardiac failure. Clin Sci.

[21] Vidlund M, Hakanson E, Friberg O, Juhl-Andersen S, Holm J, Vanky F, Sunnermalm L, Borg JO, Sharma R, Svedjeholm R (2012). GLUTAMICS–a randomized clinical trial on glutamate infusion in 861 patients undergoing surgery for acute coronary syndrome. J Thorac Cardiovasc Surg.

[22] Vanhanen I, Svedjeholm R, Hakanson E, Joachimsson PO, Jorfeldt L, Nilsson L, Vanky F (1998). Assessment of myocardial glutamate requirements early after coronary artery bypass surgery. Scand Cardiovasc J..

[23] Svedjeholm R, Hakanson E, Szabo Z (1999). Routine SvO2 measurement after CABG surgery with a surgically introduced pulmonary artery catheter. Eur J Cardiothorac Surg.

[24] Svedjeholm R, Vidlund M, Vanhanen I, Hakanson E (2010). A metabolic protective strategy could improve long-term survival in patients with LV-dysfunction undergoing CABG. Scand Cardiovasc J..

[25] Holm J, Hakanson E, Vanky F, Svedjeholm R (2011). Mixed venous oxygen saturation predicts short- and long-term outcome after coronary artery bypass grafting surgery: a retrospective cohort analysis. Br J Anaesth.

[26] Holm J, Hakanson RE, Vanky F, Svedjeholm R (2010). Mixed venous oxygen saturation is a prognostic marker after surgery for aortic stenosis. Acta Anaesthesiol Scand.

[27] Dahlin LG, Kagedal B, Nylander E, Olin C, Rutberg H, Svedjeholm R (2003). Unspecific elevation of plasma troponin-T and CK-MB after coronary surgery. Scand Cardiovasc J..

[28] Doenst T, Amorim PA (2010). Metabolic therapy in cardiac surgery—”optimizing the engine’s fuel supply and more…”. Scand Cardiovasc J..

[29] Vidlund M, Tajik B, Hakanson E, Friberg O, Holm J, Vanky F, Svedjeholm R (2016). Post hoc analysis of the glutamics-trial: intravenous glutamate infusion and use of inotropic drugs after cabg. BMC Anesthesiol..

[30] Svedjeholm R, Hakanson E, Vanhanen I (1995). Rationale for metabolic support with amino acids and glucose-insulin-potassium (GIK) in cardiac surgery. Ann Thorac Surg.

[31] Thomassen A, Nielsen TT, Bagger JP, Pedersen AK, Henningsen P (1991). Antiischemic and metabolic effects of glutamate during pacing in patients with stable angina pectoris secondary to either coronary artery disease or syndrome X. Am J Cardiol.

[32] Slogoff S, Keats AS (1985). Does perioperative myocardial ischemia lead to postoperative myocardial infarction?. Anesthesiology.

[33] Langenberg CJ, Pietersen HG, Geskes G, Wagenmakers AJ, Lange SD, Schouten HJ, Soeters PB (2001). The effect of glutamate infusion on cardiac performance is independent of changes in metabolism in patients undergoing routine coronary artery bypass surgery. Clin Sci.

[34] Young YR, Sheu BF, Li WC, Hsieh TM, Hung CW, Chang SS, Lee CC (2014). Predictive value of plasma brain natriuretic peptide for postoperative cardiac complications–a systemic review and meta-analysis. J Crit Care.

[35] Crescenzi G, Landoni G, Bignami E, Belloni I, Biselli C, Rosica C, Guarracino F, Marino G, Zangrillo A (2009). N-terminal B-natriuretic peptide after coronary artery bypass graft surgery. J Cardiothorac Vasc Anesth.

[36] Vanky F, Hakanson E, Maros T, Svedjeholm R (2004). Different characteristics of postoperative heart failure after surgery for aortic stenosis and coronary disease. Scand Cardiovasc J..

[37] Rao V, Ivanov J, Weisel RD, Ikonomidis JS, Christakis GT, David TE (1996). Predictors of low cardiac output syndrome after coronary artery bypass. J Thorac Cardiovasc Surg.

[38] Fellahi JL, Parienti JJ, Hanouz JL, Plaud B, Riou B, Ouattara A (2008). Perioperative use of dobutamine in cardiac surgery and adverse cardiac outcome: propensity-adjusted analyses. Anesthesiology.

